# Supplementary material for: Modelling Skylarks (Alauda arvensis) to Predict Impacts of Changes in Land Management and Policy: Development and Testing of an Agent-Based Model
Source: PLoS One. 2013 Jun 6;8(6):e65803. doi: 10.1371/journal.pone.0065803 (PMC3675089; doi:10.1371/journal.pone.0065803)
Supplement: Supporting Information S4 — The skylark ODdox as a zipped archive. (ZIP) [file pone.0065803.s004.zip › Skylark_ODdox/functions_func_0x64.html]

ALMaSS Skylark ODdox: Class Members - Functions


ALMaSS Skylark ODdox
 2.0


- Main Page
- Related Pages
- Classes
- Files

- Class List
- Class Index
- Class Hierarchy
- Class Members

- All
- Functions
- Variables
- Typedefs

- a
- b
- c
- d
- e
- f
- g
- h
- i
- j
- k
- l
- m
- n
- o
- p
- r
- s
- t
- u
- v
- w
- y
- z
- ~

### - d -

- DailyMortality()
  : Skylark\_Base
- DailyQueueAdd()
  : Pesticide
- DailyQueueClear()
  : Pesticide
- DailyQueueProcess()
  : Pesticide
- Date()
  : Calendar
- DayInYear()
  : Calendar
- DayLength()
  : Calendar
- DeciduousForest()
  : DeciduousForest
- DeepPlough()
  : Farm
- DefendTerritory()
  : Skylark\_Male
- Diffuse()
  : RodenticideManager
- DiffusionFunction()
  : Pesticide
- DiffusionMaskInit()
  : Pesticide
- DiffusionMaskInitTest()
  : Pesticide
- DiffusionSprayPixel()
  : Pesticide
- Diffusor()
  : Diffusor
- DisplayLocations()
  : Population\_Manager
- DistributeRing()
  : RodenticideManager
- Do()
  : Crop
- DoAfter()
  : Population\_Manager
- DoAlmostLast()
  : Population\_Manager
- DoBefore()
  : Population\_Manager
- DoDeath()
  : RodenticideManager
- DoDevelopment()
  : RoadsideVerge
  , GreenElement
  , Scrub
  , NaturalGrass
  , UnsprayedFieldMargin
  , ForestElement
  , BeetleBank
  , Orchard
  , OrchardGrass
  , OrchardBand
  , LE
  , VegElement
  , Field
  , Hedges
  , HedgeBank
- DoDiffuse()
  : RodenticideManager
- DoFirst()
  : Population\_Manager
  , Skylark\_Population\_Manager
- DoImmigration()
  : RodenticideManager
- DoIt()
  : Farm
- DoLast()
  : Population\_Manager
- DoPlaceBait()
  : RodenticideManager
- DumpAllSymbolsAndExit()
  : Landscape
  , Configurator
- DumpCentroids()
  : Landscape
- DumpMap()
  : Landscape
- DumpMapGraphics()
  : Landscape
  , SkTerritories
- DumpMapInfoByArea()
  : Landscape
- DumpPublicSymbols()
  : Configurator
  , Landscape
- DumpRodenticideMap()
  : RodenticideManager
- DumpSymbols()
  : Configurator
- DumpTreatCounters()
  : Landscape
- DumpVegAreaData()
  : Landscape
- Dying()
  : TAnimal


- Generated on Thu Jan 10 2013 13:15:39 for ALMaSS Skylark ODdox by
   1.8.1.1
